# Comparative Metabolomics of Small Molecules Specifically Expressed in the Dorsal or Ventral Marginal Zones in Vertebrate Gastrula

**DOI:** 10.3390/metabo12060566

**Published:** 2022-06-20

**Authors:** Yukako Suzuki, Ryosuke Hayasaka, Masako Hasebe, Satsuki Ikeda, Tomoyoshi Soga, Masaru Tomita, Akiyoshi Hirayama, Hiroki Kuroda

**Affiliations:** 1 Institute for Advanced Biosciences, Keio University, Tsuruoka 997-0052, Yamagata, Japan; suzuki_yukako@keio.jp (Y.S.); ryosukeh@sfc.keio.ac.jp (R.H.); mhasebe@ttck.keio.ac.jp (M.H.); satsuki@ttck.keio.ac.jp (S.I.); soga@sfc.keio.ac.jp (T.S.); mt@sfc.keio.ac.jp (M.T.); hirayama@ttck.keio.ac.jp (A.H.); 2 Systems Biology Program, Graduate School of Media and Governance, Keio University, Fujisawa 252-0882, Kanagawa, Japan

**Keywords:** metabolomic analysis, *Xenopus laevis*, dorsal–ventral patterning, Spemann organizer, early embryogenesis

## Abstract

Many previous studies have reported the various proteins specifically secreted as inducers in the dorsal or ventral regions in vertebrate gastrula. However, little is known about the effect on cell fate of small molecules below 1000 Da. We therefore tried to identify small molecules specifically expressed in the dorsal marginal zone (DMZ) or ventral marginal zone (VMZ) in vertebrate gastrula. Small intracellular and secreted molecules were detected using explants and supernatant samples. Hydrophilic metabolites were analyzed by capillary ion chromatography–mass spectrometry and liquid chromatography–mass spectrometry, and lipids were analyzed by supercritical fluid chromatography–tandem mass spectrometry. In total, 190 hydrophilic metabolites and 396 lipids were identified. The DMZ was found to have high amounts of glycolysis- and glutathione metabolism-related metabolites in explants, and the VMZ was richer in purine metabolism-related metabolites. We also discovered some hydrophilic metabolites and lipids differentially contained in the DMZ or VMZ. Our research would contribute to a deeper understanding of the cellular physiology that regulates early embryogenesis.

## 1. Introduction

In phylum Chordata, the spherical egg becomes a blastula stage (the embryo is called a blastocyst at this stage in mammals), which contains many cells with pluripotency, followed by gastrulation. Then, dynamic morphological changes in the entire embryo occur, shaping the unique morphology of the species. To understand the early developmental stages in phylum Chordata, the approach often chosen is to focus on three axes such as anterior–posterior, dorsal–ventral, and left–right directions and to elucidate the molecular mechanisms of each [1].

The African clawed frog (*Xenopus laevis*) is frequently used as an experimental model organism to elucidate the molecular mechanisms that control early development [2]. A number of studies have used this experimental animal to focus on the anterior–posterior and left–right axes, but many more studies have focused on the dorsal–ventral axis. In this animal, during fertilization, the dorsal–ventral axis is determined by the rotation of the surface layer starting from the point of sperm entry. The dorsal determinant, which was present on the surface layer on the vegetal side of the egg, moves on microtubules to the opposite side of the sperm entry point [3,4]. In other words, the dorsal–ventral axis is already determined at the one-cell stage. At the gastrula stage, part of the dorsal region where the dorsal determinant was present gives rise to a region called the Spemann organizer [5]. The Spemann organizer is a region that was first described by Spemann and Mangold in 1924 [6]. They showed that when the dorsal region of an amphibian gastrula embryo is transplanted to its opposite side, the ventral region, a secondary axis containing neural tubes, notochord, and somites, is formed. This study provides the first evidence to support the idea that there is an induction center in early development that exerts a major influence on developmental fate by acting on surrounding regions.

In the 1990s, many screening approaches comparing the genes expressed in the dorsal and ventral regions in *Xenopus laevis* gastrula embryos (*Xenopus* gastrula) found molecules that are expressed and have organizer-specific induction activity in Spemann organizers. For example, Nodal-related proteins are ligand molecules that bind to specific receptors. In contrast, various antagonist molecules have been found such as Chordin, Noggin, and Follistatin, which are antagonists of bone morphogenetic proteins (BMPs); Frizzled-related proteins, secreted Frizzled-related proteins (sFRPs), and Dickkopf, which are antagonists of Wnts; and Lefty/Antivin, which is an antagonist of Nodal-related proteins [7,8,9,10,11,12]. In addition to the above, many other molecules have been found to be expressed specifically on the dorsal or ventral sides [2].

However, most of the molecules reported previously are peptide-based, and reports of small molecules (<1000 Da) are extremely limited. However, protein is only one of the five macronutrients, and non-peptide molecules are included in various hormones and neurotransmitters in the adult body. Therefore, we assume that small non-peptidic molecules are a non-negligible part of the early developmental stages because some small molecules have been identified that have a significant effect on morphogenesis during embryogenesis. For instance, serotonin (MW 176), expressed during the cleavage stage, is involved in the determination of the left–right axis [13]; retinoic acid (MW 300) and adrenaline (MW 183), expressed from gastrula to neurula, are required for posteriorization [14,15]; and *γ*-aminobutyric acid (GABA) (MW 103), expressed after neurula, has a role in embryonic elongation [16]. Research on metabolic activities via metabolomics in the embryo holds great potential to raise our understanding of the developmental processes that control cell-type specification by combining with the other omics data obtained by previous studies [17,18,19]. It is also significant to find dorsoventral-specific secreted molecules, generally contained in the supernatant of cultured cells, and small molecules that determine cell-autonomous differentiation inside cells. In fact, some previous studies using metabolomic analysis have reported metabolic activity differences between dorsal and ventral cells in *Xenopus* embryos during the cleavage cycles [20,21,22]. However, to our knowledge, there is no study focusing on *Xenopus* gastrula.

In this study, we therefore carried out metabolomic analyses to identify small molecules specifically contained in the dorsal or ventral regions in vertebrate gastrula. To detect both small molecules expressed in the dorsal marginal zone (DMZ), which includes the area of the Spemann organizer, or expressed in the ventral marginal zone (VMZ) and secreted from the DMZ or VMZ, capillary ion chromatography–mass spectrometry (capillary IC-MS), liquid chromatography–mass spectrometry (LC-MS), and supercritical fluid chromatography–tandem mass spectrometry (SFC-QqQMS) were performed using two types of samples: explants and supernatants. We found some dorsoventral-specific small molecules in *Xenopus* gastrula.

## 2. Results

### 2.1. Analyses of Hydrophilic Metabolites

#### 2.1.1. In Total, 186 and 98 Hydrophilic Metabolites Were Identified in Explants and Supernatants, Respectively

**Figure 1:**
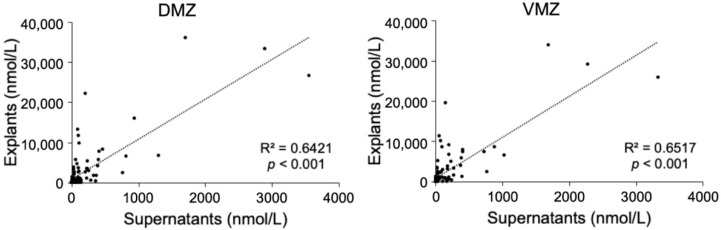
Correlations of the hydrophilic metabolites in explants and supernatants.

This study sought to identify the metabolites specifically contained in the DMZ or VMZ in *Xenopus* gastrula. To detect them comprehensively, metabolomic analyses were carried out using explants and supernatants. First, we performed a hydrophilic metabolome analysis by capillary IC-MS and LC-MS, resulting in the detection of 186 and 98 metabolites in explants and supernatants, respectively. Of these, 94 metabolites were common to both samples. Ninety-two metabolites, including amino acids such as His, Met, Ser, and Trp as well as deoxynucleotides, were unique to explants. In contrast, four metabolites (2-oxoadipic acid, diethanolamine, hexylamine, and *N*-acetylmuramic acid) were unique to supernatants. Scatter plots of the 94 hydrophilic metabolites that were common to explants and supernatants showed a significant correlation between the two types of samples (DMZ, R^2^ = 0.6421, *p* < 0.001; VMZ, R^2^ = 0.6517, *p* < 0.001; Figure 1).

#### 2.1.2. The DMZ and the VMZ Had, Respectively, High Amounts of Glycolysis- and Glutathione Metabolism-Related Metabolites and Purine Metabolism-Related Metabolites in Explants

**Figure 2:**
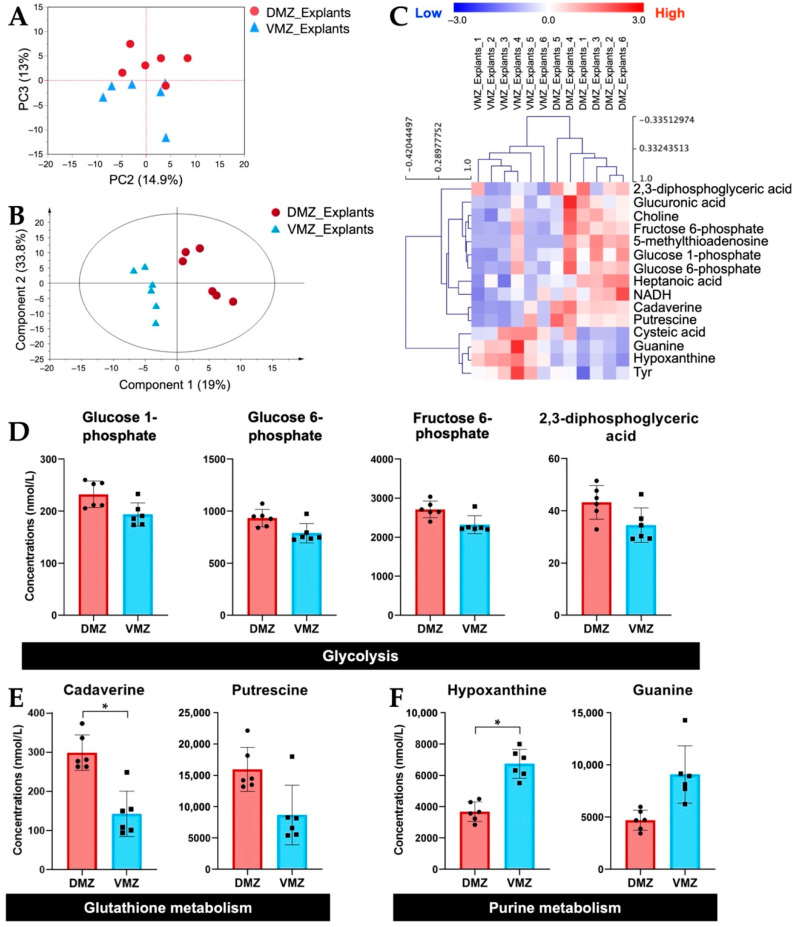
Identification of hydrophilic metabolites differentially expressed between the DMZ and VMZ in explants (DMZ and VMZ explants). (**A**) Principal component analysis (PCA) score visualizing the relationship between the DMZ (red circle) and VMZ (blue triangle) explants using hydrophilic metabolites. The contribution ratios were 14.9% and 13% for PC2 and PC3, respectively. (**B**) Partial least squares–discriminant analysis (PLS-DA) score plots for the DMZ (red circle) and VMZ (blue triangle) explants. (**C**) Hierarchical clustering analysis (HCA) for the top 15 metabolites by variable importance in projection (VIP) scores in explants. Rows display the metabolite, and columns represent the sample. Metabolites with relatively low contents are displayed in blue, whereas metabolites with relatively high contents are displayed in red. The brightness of each color corresponds to the magnitude of the difference compared with the mean value. The number at the end of each sample name corresponds to samples made from the same embryos. (**D**–**F**) Comparison of the concentrations of metabolites that differed notably between the DMZ and VMZ explants. Symbols represent each sample, and the bar graph indicates the mean ± SD (*n* = 6, each sample from 20 explants). * FDR adjusted *p* < 0.05 (Welch’s *t*-test, Benjamini–Hochberg procedure). (**D**) Bar graphs of the metabolites involved in glycolysis in the top 15 metabolites by VIP scores. (**E**) Bar graphs of the metabolites involved in glutathione metabolism in the top 15 metabolites by VIP scores. (**F**) Bar graphs of the metabolites involved in purine metabolism in the top 15 metabolites by VIP scores.

Next, we investigated the differences in the amounts of hydrophilic metabolites in the DMZ and VMZ in each sample. For the purpose of direct visual comparison, the *z*-score normalized data of hydrophilic metabolites in the DMZ and VMZ in the explants (DMZ and VMZ explants) were analyzed by unsupervised principal component analysis (PCA). As a result, the DMZ and VMZ groups were separated by principal component 3 (Figure 2A). Hence, to discover the metabolites that have an influence on the differences in the DMZ and VMZ explants, partial least squares-discriminant analysis (PLS-DA) was performed, and variable importance in projection (VIP) scores were calculated (Figure 2B). Explants samples were classified into the DMZ and VMZ groups by hierarchical clustering analysis (HCA) using the top 15 metabolites by VIP scores (Figure 2C). Of these metabolites, 2,3-diphosphoglyceric acid, 5-methylthioadenosine, cadaverine, choline, fructose 6-phosphate, glucose 1-phosphate, glucose 6-phosphate, glucuronic acid, heptanoic acid, NADH, and putrescine were more abundant in the DMZ, whereas cysteic acid, guanine, hypoxanthine, and Tyr were more abundant in the VMZ. The HCA results indicated that the DMZ had high amounts of glycolysis- and glutathione metabolism-related metabolites and the VMZ had high purine metabolism-related metabolites in explants (Figure 2D–F). These metabolites highly contributed to the discrimination between the DMZ and VMZ explants. Further analysis found that not only the metabolites shown in Figure 2D but most of the other metabolites involved in glycolysis were more active in the DMZ than in the VMZ (Supplementary Figure S1). The concentrations of cadaverine and hypoxanthine were also significantly different between the DMZ and VMZ explants (FDR adjusted *p* < 0.05). The results obtained by gas chromatography–mass spectrometry (GC-MS) also demonstrated that hypoxanthine and guanine were specifically contained in the VMZ explants (Supplementary Figure S2).

#### 2.1.3. Hypoxanthine, Guanine, and Glucuronic Acid Were Differentially Expressed between the DMZ and VMZ in the Two Types of Samples

**Figure 3:**
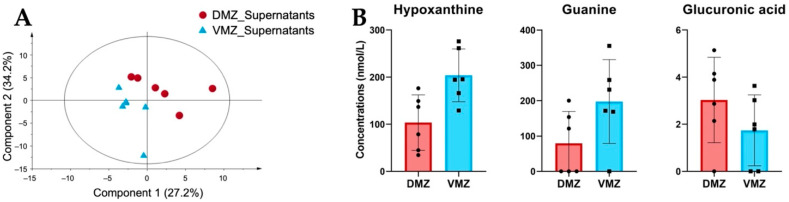
Comparative analysis of the characteristic hydrophilic metabolites identified in supernatants. (**A**) PLS-DA score plots for the supernatants of the DMZ (red circle) and VMZ (blue triangle) samples (DMZ and VMZ supernatants) based on hydrophilic metabolite data. (**B**) Comparison of hypoxanthine, guanine, and glucuronic acid concentrations in the DMZ and VMZ supernatants. Symbols represent each sample and the bar graph indicates the mean ± SD (*n* = 6, each sample from 20 explants).

Differences in the levels of hydrophilic metabolites in the DMZ and VMZ explants are shown in Figure 2. We therefore carried out PLS-DA to investigate the characteristics of hydrophilic metabolites in the supernatants of the DMZ and VMZ samples (DMZ and VMZ supernatants) (Figure 3A). The top 15 metabolites by VIP scores in supernatants were selected similarly to explants, and consequently, the DMZ contained relatively higher concentrations of Ala, *β*-ala, ATP, betaine, carnitine, citric acid, CTP, diethanolamine, glucuronic acid, and GTP. In contrast, the VMZ contained relatively more 1-methylhistamine, *α*-methylserine, cysteine sulfinic acid, guanine, hypoxanthine, and pyruvic acid compared to the DMZ (Supplementary Table S1). Of these, hypoxanthine, guanine, and glucuronic acid were included in the top 15 metabolites by VIP scores in explants as well (Figure 3B).

### 2.2. Lipidomic Analyses

**Figure 4:**
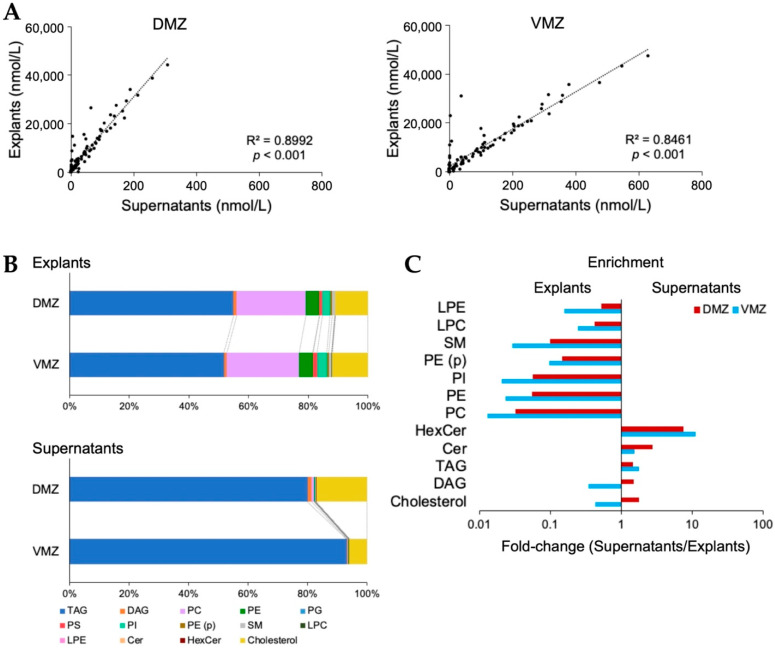
Lipid characteristics between the DMZ and VMZ in explants and supernatants. (**A**) Correlations of the lipids detected between explants and supernatants. (**B**) Bar graphs showing the proportion of each lipid class in the total lipid content. (**C**) Enrichment of lipid classes in explants and supernatants. The *x*-values are the ratios obtained by dividing the proportion in supernatants by that in explants. Any lipid class whose value was zero was excluded. TAG, triacylglycerol; DAG, diacylglycerol; PC, phosphatidylcholine; PE, phosphatidylethanolamine; PG, phosphatidylglycerol; PS, phosphatidylserine; PI, phosphatidylinositol; PE (p), alkenyl-acyl phosphatidylethanolamine; SM, sphingomyelin; LPC, lysophosphatidylcholine; LPE, lysophosphatidylethanolamine; Cer, ceramide; HexCer, hexosylceramides.

For further investigation, analyses of lipids in the explants and supernatants were conducted using SFC-QqQMS. In total, 396 and 95 lipids belonging to 14 lipid classes were detected in explants and supernatants, respectively. The 301 lipids identified only in explants contained diacylglycerol (DAG), phosphatidylethanolamine (PE), phosphatidylcholine (PC), triacylglycerol (TAG), and alkenyl-acyl phosphatidylethanolamine (PE (p)). Moreover, all 95 lipids detected in supernatants overlapped with those in explants, and their levels were adequately correlated (DMZ, R^2^ = 0.8992, *p* < 0.001; VMZ, R^2^ = 0.8461, *p* < 0.001; Figure 4A).

Next, we evaluated the relationship between the content of each lipid class in explants and supernatants. The contents of TAG (DMZ, 54.9%; VMZ, 51.8%) and PC (DMZ, 23.3%; VMZ, 24.2%) were relatively higher in explants, whereas that of TAG (DMZ, 80.1%; VMZ, 93.2%) was higher in supernatants (Figure 4B). A comparison of the proportion of each lipid class in the total lipid content showed that the amounts of hexosylceramide (HexCer) (DMZ, 7.7 times; VMZ, 11 times) and ceramide (Cer) (DMZ, 2.7 times; VMZ, 1.6 times) were more than 1.5 times in supernatants than in explants. However, the levels of PC (DMZ, 0.032 times; VMZ, 0.013 times), PE (DMZ, 0.055 times; VMZ, 0.023 times), phosphatidylinositol (PI) (DMZ, 0.056 times; VMZ, 0.021 times), and sphingomyelin (SM) (DMZ, 0.10 times; VMZ, 0.029 times) were less than one-tenth of their levels in supernatants than in explants (Figure 4C). Interestingly, the ratio of DAG (DMZ, 1.5 times; VMZ, 0.35 times) and cholesterol (DMZ, 1.8 times; VMZ, 0.44 times) was markedly different between the DMZ and VMZ. Finally, the lipids detected in the DMZ or VMZ regions were examined. In supernatants, 10 and 17 lipids were identified only in the DMZ or VMZ, respectively (Table 1).

## 3. Discussion

Single-cell capillary electrophoresis electrospray ionization (CE-ESI)-MS has revealed metabolic activity differences between dorsal and ventral cells in live *Xenopus* embryos during the cleavage cycles [20,21,22]. Those studies confirmed the effect of metabolic changes on cell differentiation. In the present study, we identified small molecules in the gastrula period, at stage 10, and succeeded in quantifying 190 hydrophilic metabolites using explants and supernatants. A previous report detected a core set of 48 metabolites in nearly all of their experiments, including amino acids, nucleotides, and phosphorylated sugars, in single *Xenopus* eggs or embryos from stages 0 to 9 [23]. Of these 48, we could confirm the detection of 41 metabolites, except for three that were not included in our laboratory’s library. The remaining four metabolites, taurine, NAD+, NADP+, and acetyl-CoA, were not detected in this study.

The DMZ was found to contain higher amounts of glycolysis- and glutathione metabolism-related metabolites in explants (Figure 2D,E, Supplementary Figure S1). Glycolysis-dependent intracellular pH controls Wnt signaling in the tail bud, which promotes the fate of paraxial mesoderm from neuromesodermal progenitors required for sustained axial elongation [24,25]. Given this fact, the glycolytic activity could affect dorsalization via Wnt signaling in the gastrula. Additionally, in a previous study [22], the midline dorsal-animal cells in 16-cell *Xenopus* embryos were noticeably richer in Ser, Gly, Thr, and urocanic acid than the midline ventral-animal cells, whereas the ventral regions were richer in hypoxanthine compared to the dorsal regions. In the gastrula, there was no difference in the activity of the Ser–Gly–Thr pathway between the DMZ and VMZ. This might be because remodeling of core metabolic pathways occurred during the mid-blastula transition (MBT) [23]. However, interestingly, more hypoxanthine was significantly accumulated in the VMZ than the DMZ even in the gastrula, a stage after the MBT (Figure 2F and Figure 3B). Our analyses uncovered that hypoxanthine, guanine, and glucuronic acid were differentially active between the DMZ and VMZ in *Xenopus* gastrula (Figure 2C,F and Figure 3B). Further investigation is necessary to conclude whether these metabolites are able to define cell fates.

Lipid profiling during early vertebrate development has been reported along with metabolomics data [26,27,28,29,30], and it is known that PC 18:0–22:6, PE 16:0–20:4, and SM d18:1–16:0 are, respectively, enriched in the retina, pharynx, and notochord [29]. In this work, we succeeded in identifying 396 lipids in *Xenopus* gastrula using two types of samples. In supernatants, the ratio of each lipid class/the total lipid content was higher for HexCer and Cer than that in explants; but the ratio of PC, PE, PI, and SM in explants was remarkably higher than that in supernatants (Figure 4C). A previous study reported that tissues contained a large family of PC, and cavities that form the future gut and neural crest contained abundant Cer in stage-13 and stage-19 *Xenopus* embryos [31]. The cavities would be similar in composition to the supernatant samples used in this work because they would be rich in secreted substances released from cells rather than substances derived from tissues. Given this assumption, our data support the results of the previous report. In addition, our study discovered some lipids specifically contained in the DMZ or VMZ in *Xenopus* gastrula (Table 1). These lipids can be molecular markers in the dorsal or ventral regions.

In this study, supernatant samples were used to reduce the influence of small intracellular molecules and to detect secreted small molecules. Explants had to be cultured for 60 min to detect trace amounts of secreted small molecules, although it should be noted that the metabolites in the supernatant may have been altered during incubation. Additionally, we could not determine whether the small molecules identified in our metabolomic analyses affected the phenotypes or were just the results of cell differentiation in the DMZ and VMZ. Therefore, it is necessary to conduct future functional experiments using the candidate metabolites, which were specifically expressed in the DMZ or VMZ.

In conclusion, we identified 186 and 98 hydrophilic metabolites and 396 and 95 lipids in explants and supernatants, respectively, using a combination of capillary IC-MS, LC-MS, and SFC-QqQMS. Glycolysis- and glutathione metabolism-related metabolites were more active in the DMZ explants, whereas purine metabolism-related metabolites were more active in the VMZ explants. As a result of comparing the data between explants and supernatants, hypoxanthine, guanine, and glucuronic acid were differentially contained between the DMZ and VMZ in the two types of samples. Furthermore, dorsoventral-specific lipids were found. To our knowledge, this is the first study to show large metabolite data for small molecules in *Xenopus* gastrula. Our research would contribute to a deeper understanding of the cellular physiology that regulates early embryogenesis.

## 4. Materials and Methods

### 4.1. Embryo Manipulations

*Xenopus* embryos were obtained by in vitro fertilization. The details of in vitro fertilization are described in Ohata et al., 2014 [32]. Twenty DMZs or VMZs per sample were dissected at stage 10 in 1× SS (Steinberg’s solution). 1× SS was prepared by diluting 10× SS to a concentration of one-tenth with ultrapure water, adding 0.1 g/L of kanamycin sulfate (Sigma-Aldrich, St. Louis, MO, USA), and then autoclaving. 10× SS contained 580 mmol/L NaCl, 6.7 mmol/L KCl, 3.4 mmol/L Ca(NO_3_)_2_, 8.3 mmol/L MgSO_4_, and 0.1 g/L kanamycin sulfate and was adjusted to pH 7.4 using sodium hydroxide. Dissected samples were washed with 0.5× SS, and most of the yolk-rich regions were removed. 0.5× SS was prepared by diluting 10× SS to a concentration of one-twentieth with ultrapure water. The DMZs and VMZs were each put into 100 μL of 0.5× SS in 1.5 mL siliconized tubes and cultured for 60 min at 22 °C. After that, 50 μL of the supernatant was collected as a supernatant sample. Samples of explants or supernatants were immediately stored at –80 °C (*n* = 6). In a 1.5 mL siliconized tube, 50 μL of 0.5× SS, an uncultured medium was additionally used as the blank of supernatant samples (*n* = 6). It was also stored at –80 °C after placing it at 22 °C for 60 min to keep the same condition.

### 4.2. Extraction of Hydrophilic Metabolites and Lipids

For metabolite extraction, 1 mL of the mixture of methanol (FUJIFILM Wako Pure Chemicals Corp., Osaka, Japan), chloroform (FUJIFILM Wako Pure Chemicals Corp.), and milli-Q water (Merck KGaA, Gernsheim, Germany) (10:5:3) containing internal standards (IS) was added to each sample. Five microliters of IS for hydrophilic metabolites was composed of 100 µmol/L methionine sulfone (FUJIFILM Wako Pure Chemicals Corp.), tryptophan-^13^C_11_, ^15^N_2_ (TAIYO NIPPON SANSO Corp., Tokyo, Japan), and camphor 10-sulfonic acid (FUJIFILM Wako Pure Chemicals Corp.). For lipids, 5 μL of IS comprised the mouse SPLASH^®^ LIPIDOMIX^®^ Mass Spec Internal Standard (Avanti Polar Lipids, Alabaster, AL, USA) and the internal standard mix. The details of the composition are described previously [33]. 

After using a vortex mixer for 1 min, the solution was moved to a 1.5 mL microcentrifuge tube. It was sonicated for 5 min and centrifuged (16,000× *g*, 5 min, 4 °C). Eight hundred microliters of the upper layer was transferred to a 2 mL microcentrifuge tube, and then the supernatant was mixed well with 220 μL of chloroform and milli-Q water followed by centrifugation (16,000× *g*, 3 min, 4 °C). Five hundred microliters of the aqueous layer and 400 μL of the organic layer were, respectively, transferred to new tubes. The subsequent steps, including dryness, were conducted as previously described [33].

### 4.3. Analyses of Hydrophilic Metabolites

The analytical methods of anionic metabolites by capillary IC-MS were the same as those represented in Hirayama et al., 2020 [34]. Cationic metabolites were measured using LC-MS according to previously described methods with some modifications [35]. Briefly, LC-MS was performed using an Agilent 1290 Infinity LC system (Agilent Technologies, Santa Clara, CA, USA) equipped with a Q Exactive Orbitrap MS system (Thermo Fisher Scientific, Waltham, MA, USA). Separation of cationic metabolites was performed on a HILIC-Z column (150 × 2.1mm, 2.7 μm; Agilent Technologies). The injection volume was 1 µL, and the column temperature was maintained at 40 °C. The mobile phase consisted of 20 mmol/L ammonium formic acid + 0.25% (*v/v*) formic acid (A) and 20 mmol/L ammonium formic acid + 0.25% formic acid in 90% (*v/v*) acetonitrile (B). The eluent flow rate was 0.25 mL/min, and the gradient was as follows: 0–15 min, 100% to 70% B; 15–20 min, 70% to 10% B; 20–23 min, 10% B; and 23–30 min, 100% B. The Q Exactive mass spectrometer was operated in a heated ESI (HESI) positive-ion mode using the following source parameters: auxiliary gas temperature = 300 °C, auxiliary gas flow rate = 10 (arbitrary units), spray voltage = 3.5 kV, capillary temperature = 250 °C, sheath gas flow rate = 40 (arbitrary units), and S-lens = 35 (arbitrary units). The acquisition method combined two scan events corresponding to a full MS scan mode and a parallel reaction monitoring (PRM) mode. The parameters in full MS scan mode were as follows: resolution, 35,000; auto gain control target, 3.0 × 10^6^; maximum ion injection time, 200 ms; and scan range, 50–750 *m/z*. Additionally, the parameters in PRM mode were as follows: resolution, 17,500; auto gain control target, 2 × 10^5^; maximum ion injection time, 100 ms; inclusion *m/z* list: 104.0706 (GABA), 118.0863 (Val), 132.1019 (Leu, Ile), 166.0863 (Phe), and 182.0482 (methionine sulfone).

### 4.4. Lipidomic Analyses

Lipids were analyzed by SFC-QqQMS based on previous studies [33,36]. Briefly, SFC-QqQMS experiments were conducted using an Agilent 1260 Infinity II SFC system equipped with an Agilent 6470A triple quadrupole LC/MS system with an Agilent jet stream (AJS) ESI interface. Lipids were separated on an ACQUITY UPC2 Torus DEA column (3.0 × 100 mm, 1.7 µm; Waters Corp., Milford, MA, USA). Lipid classes measured were as follows: Cer, cholesterol, DAG, HexCer, lysophosphatidylcholine (LPC), lysophosphatidylethanolamine (LPE), phosphatidic acid (PA), PC, alkyl-acyl phosphatidylcholine (PC (e)), alkenyl-acyl phosphatidylcholine (PC (p)), PE, PE (p), phosphatidylglycerol (PG), PI, phosphatidylserine (PS), SM, and TAG. The details are previously described [33]. Dynamic multiple reaction monitoring (MRM) transition based on the previous in-house lipid MRM library was used for the lipidomic analyses [36].

### 4.5. Data Analyses

The raw data obtained by capillary IC-MS and LC-MS were analyzed using the TraceFinder software (version 5.0, Thermo Fisher Scientific). MassHunter software (version 10.0, Agilent Technologies) was also used to analyze the data acquired by SFC-QqQMS. To remove all the peaks coming from the background, the amounts of metabolites in explants were calculated by subtracting the mol of individual metabolites identified in the technical blank, which were similarly pretreated and analyzed. The amounts of metabolites in supernatants were also calculated by subtracting those in the 0.5× SS sample. The negative values were changed to zero. The concentration when dissolved in 50 µL of water was used in an explants sample to match the volume of a supernatant sample. The data on metabolites detected in more than half of the samples were used (*n* = 6). Statistical significance was determined using an FDR adjusted *p*-value (Welch’s *t*-test, Benjamini-Hochberg procedure). PCA, PLS-DA, and HCA were, respectively, analyzed using JMP (version 15.0), SIMCA (version 13.0), and MeV (version 10.2). The *z*-score normalized data were used for all multivariate analyses, and PCA, PLS-DA, and HCA were, respectively, performed using a covariance matrix, the default SIMCA cross-validation procedure, and Pearson’s correlation coefficient. Excel (version 16.58) was used for multiple correlation coefficients (Pearson’s correlation coefficient, Student’s *t*-test). Bar graphs were made using GraphPad Prism (version 8.4.3) and Excel.

## Figures and Tables

**Table 1:** Lipids detected only in the supernatants of the DMZ or VMZ samples.

Lipid Class	DMZ	VMZ
Triacylglycerol	TAG 56:2	TAG 48:4
		TAG 48:5
		TAG 62:10
		TAG 64:12
		TAG 64:13
		TAG 64:17
Phosphatidylcholine		PC 16:0–20:5
Phosphatidylethanolamine	PE 16:0–22:6	
Phosphatidylinositol		PI 18:0–20:5
		PI 18:0–22:6
Alkenyl-Acyl Phosphatidylethanolamine		PE 16:1p–22:6
		PE 18:1p–20:5
		PE 18:2p–22:6
Sphingomyelin	SM d18:1–22:1	SM d18:1–18:2
Lysophosphatidylcholine	LPC 16:0	LPC 18:2
	LPC 22:0	
Lysophosphatidylethanolamine	LPE 16:0	LPE 22:6
	LPE 18:0	
	LPE 20:5	
Ceramide	Cer d18:1–22:0	Cer d18:1–16:0
		Cer d18:1–18:2
Cholesterol	Cholesterol	

## Data Availability

Data are contained in the article and supplementary materials.

## Supplementary Materials

The following supporting information can be downloaded at https://www.mdpi.com/article/10.3390/metabo12060566/s1, S1. Materials and Methods. Figure S1: Comparison of the concentrations of metabolites involved in glycolysis and pentose and glucuronic acid interconversion pathways in explants. Figure S2: Identification of metabolites differentially expressed between the DMZ and VMZ in explants (DMZ and VMZ explants) using GC-MS. Table S1: List of hydrophilic metabolites with high VIP scores (VIP scores >1.0) in supernatants. Table S2: List of hydrophilic metabolites with high VIP scores (VIP scores >1.0) in explants.
