# Supplementary material for: Comparative Metabolomics of Small Molecules Specifically Expressed in the Dorsal or Ventral Marginal Zones in Vertebrate Gastrula
Source: Metabolites. 2022 Jun 20;12(6):566. doi: 10.3390/metabo12060566 (PMC9229639; doi:10.3390/metabo12060566)
Supplement: Supplementary file 1 [file metabolites-12-00566-s001.zip › Supplementary Materials_Figures S1-S2.pdf]

Supporting information

Comparative Metabolomics of Small Molecules Specifically Expressed in the Dorsal or Ventral Marginal Zones in Vertebrate Gastrula

S1. Materials and Methods

1.1. Sample Preparation and Preprocessing for Gas Chromatography-Mass Spectrometry (GC-MS) Analyses

Methods of sample preparation for GC-MS analyses were the same as those mentioned in *4.1*. However, DMZs and VMZs were cultured for 90 min at 22 °C. Explants and supernatant samples (*n* = 3) were respectively mixed with 500 μL and 400 μL of methanol containing 100 μmol/L internal standards. Ribitol (FUJIFILM Wako Pure Chemical Corp., Osaka, Japan) and 2-isopropylmalic acid (Sigma-Aldrich, St. Louis, MO, USA) were used as internal standards. The explants sample was sonicated for 10 min. After using a vortex mixer for 1 min, samples were centrifuged (500× *g*, 1 min, 25 °C). Five hundred microliters of chloroform and 200 μL of milli-Q water were added to each sample, followed by centrifugation (20,000× *g*, 15 min, 4 °C). Five hundred microliters of the aqueous layer was transferred to a new tube. The supernatant was dried using a centrifugal concentrator.

Ten microliters of 20 mg/mL methoxyamine hydrochloride (FUJIFILM Wako Pure Chemical Corp.) in pyridine (FUJIFILM Wako Pure Chemical Corp.) was then added to dried samples, vortex-mixed, and centrifuged (500× *g*, 1 min, 25 °C). After incubation (90 min, 37 °C), 45 μL of MSTFA+1%TMCS (Thermo Fisher Scientific, Waltham, MA, USA) was added, vortex-mixed, and centrifuged (500× *g*, 1 min, 25 °C). The supernatant was transferred to a brown glass vial following incubation (30 min, 37 °C).

1.2. GC-MS Analyses

GC-MS was performed using an Intuvo 9000GC-5977B MSD (Agilent Technologies, Santa Clara, CA, USA). A DB-5ms Intuvo GC column (30 m × 0.25 mm, 0.25 μm; Agilent Technologies) and a Precision Hydrogen Trace 500 cc for Agilent (Peak Scientific Instruments Ltd., UK) flowing at 1.3 mL/min were used. The pulsed split mode (3:1) was used for the injection, and the volume was 2 μL. The temperature of the GC inlet and the guard chip was set at 250 °C. The GC oven temperature was programmed from 60 °C (hold times: 0,5 min) to 325 °C at a rate of 20 °C/min. The final hold time was 5 min. The temperature of the transfer line was set at 290 °C. Data were acquired over a scan range of 50–600 *m*/*z* under electron ionization (EI) at 70 eV after the solvent delay at 3.3 min.

1.3. Data Processing for GC-MS Data

The data acquired by GC-MS were analyzed using MassHunter Workstation software (ver.B.09.00, Agilent Technologies). Relative peak area (RelArea), the peak area relative to the peak areas of internal standards, was calculated for each peak to reflect the metabolite level in the samples. The amounts of metabolites identified in the technical blank and 0.5× SS sample were subtracted from those identified in explants and supernatant samples, respectively. The negative values were changed to zero. The data on metabolites detected in more than half of the samples were used (*n* = 3).

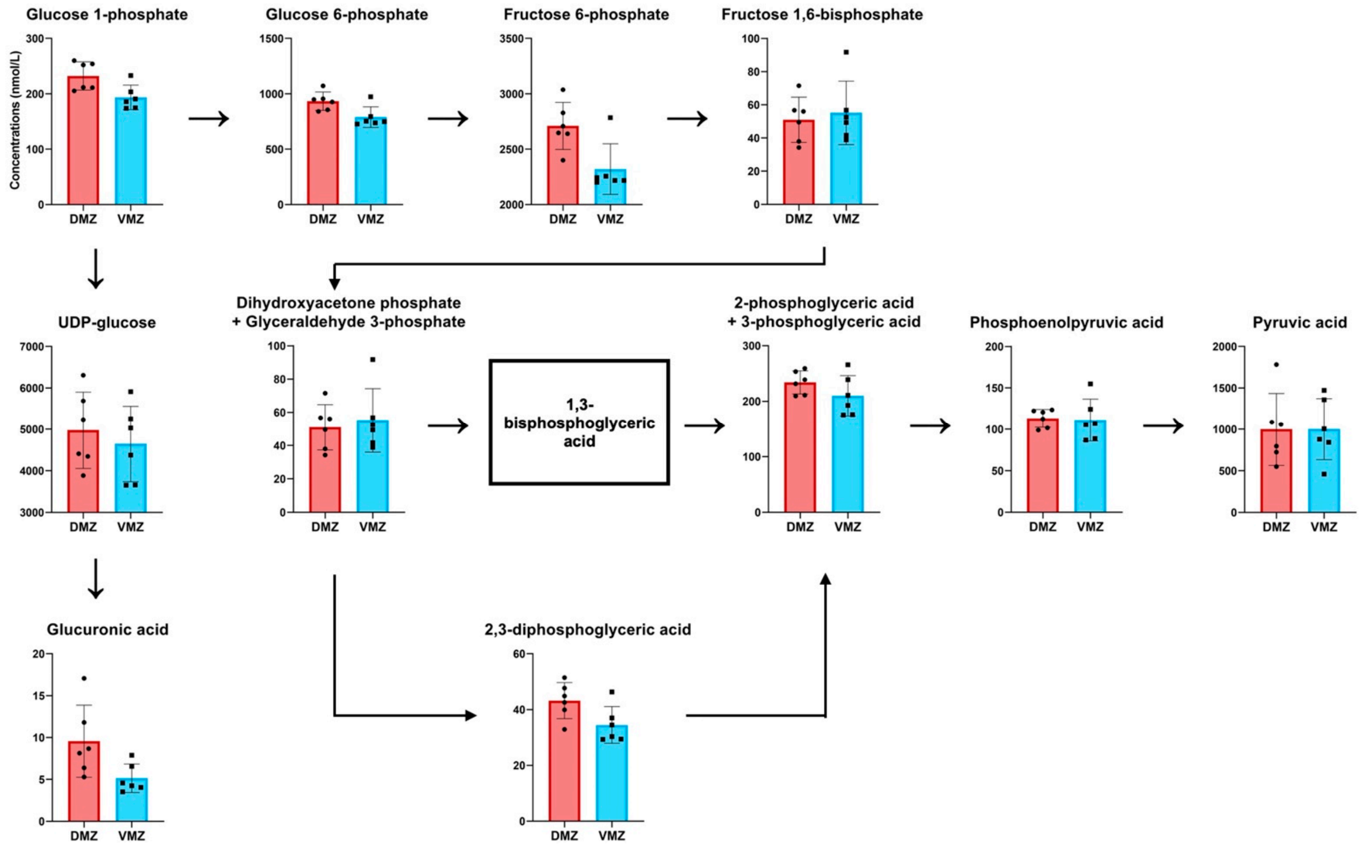

Figure S1. Comparison of the concentrations of metabolites involved in glycolysis and pentose and glucuronic acid interconversion pathways in explants. Symbols represent each sample, and the bar graph indicates the mean ± SD (n = 6, each sample from 20 explants). 1,3-bisphosphoglyceric acid was not included in our laboratory's library.

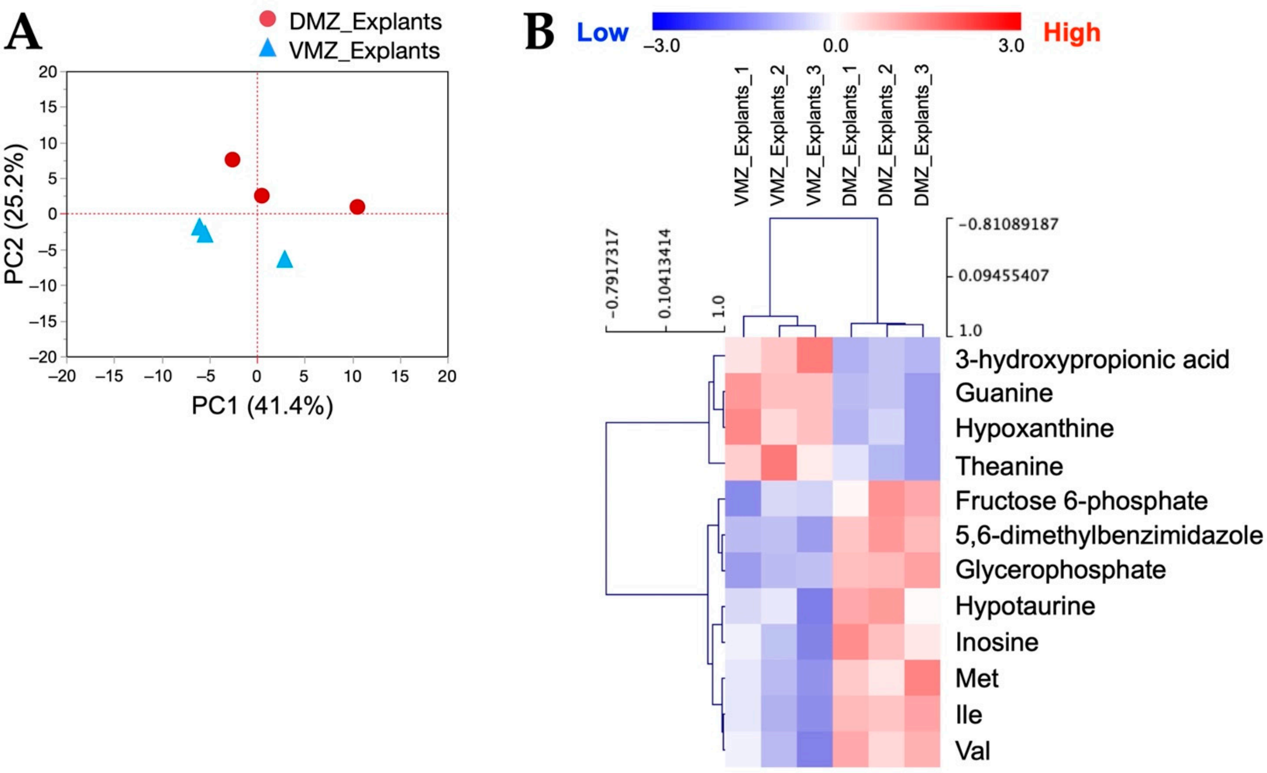

Figure S2. Identification of metabolites differentially expressed between the DMZ and VMZ in explants (DMZ and VMZ explants) using GC-MS. (**A**) PCA score visualizing the relationship between the DMZ (red circle) and VMZ (blue triangle) explants. The contribution ratios were 41.4% and 25.2% for PC1 and PC2, respectively. (**B**) HCA for the 12 metabolites of which the content was obviously different between the DMZ and VMZ explants. Rows display the metabolite, and columns represent the sample. Metabolites with relatively low contents are displayed in blue, while metabolites with relatively high contents are displayed in red. The brightness of each color corresponds to the magnitude of the difference when compared with the mean value. The number at the end of each sample name corresponds to samples made from the same embryos. $p < 0.05$ calculated by Welch's t-test, $n = 3$.
